# Chronic treatment with a smart antioxidative nanoparticle for inhibition of amyloid plaque propagation in Tg2576 mouse model of Alzheimer’s disease

**DOI:** 10.1038/s41598-017-03411-7

**Published:** 2017-06-19

**Authors:** Phetcharat Boonruamkaew, Pennapa Chonpathompikunlert, Long Binh Vong, Sho Sakaue, Yasushi Tomidokoro, Kazuhiro Ishii, Akira Tamaoka, Yukio Nagasaki

**Affiliations:** 1 Department of Materials Sciences, Graduate School of Pure and Applied Sciences, University of Tsukuba, Tennoudai 1-1-1, Tsukuba, Ibaraki 305-8573 Japan; 2 Institute of Clinical Medicine, Department of Neurology, University of Tsukuba, Tennoudai 1-1-1, Tsukuba, Ibaraki 305-8575 Japan; 3 Master’s School of Medical Sciences, Graduate School of Comprehensive Human Sciences, Tennoudai 1-1-1, Tsukuba, Ibaraki 305-8573 Japan; 4 Satellite Laboratory, International Center for Materials Nanoarchitechtonics (WPI-MANA), National Institute for Materials Sciences (NIMS), University of Tsukuba, Tennoudai 1-1-1, Tsukuba, Ibaraki 305-8573 Japan; 5 School of Pharmacy, Walailak University, Thasala, Nakhon Si Thammarat 80161 Thailand; 6 College of Alternative Medicine, Chandrakasem Rajabhat University, 39/1 Ratchadaphisek Road, Khwaeng Chantharakasem, Chatuchak Districk, Bangkok, 10900 Thailand; 7 Department of Biochemistry, Faculty of Biology and Biotechnology, University of Science, Vietnam National University Ho Chi Minh City (VNU-HCM), Ho Chi Minh City, 702500 Vietnam

## Abstract

The present study aimed to assess whether our newly developed redox nanoparticle (RNP^N^) that has antioxidant potential decreases Aβ levels or prevents Aβ aggregation associated with oxidative stress. The transgenic Tg2576 Alzheimer’s disease (AD) mice were used to investigate the effect of chronic ad libitum drinking of RNP^N^ solution for 6 months, including memory and learning functions, antioxidant activity, and amyloid plaque aggregation. The results showed that RNP^N^-treated mice had significantly attenuated cognitive deficits of both spatial and non-spatial memories, reduced oxidative stress of lipid peroxide, and DNA oxidation. RNP^N^ treatment increased the percent inhibition of superoxide anion and glutathione peroxidase activity, neuronal densities in the cortex and hippocampus, decreased Aβ(1-40), Aβ(1-42) and gamma (γ)-secretase levels, and reduced Aβ plaque observed using immunohistochemistry analysis and thioflavin S staining. Our results suggest that RNP^N^ may be a promising candidate for AD therapy because of its antioxidant properties and reduction in Aβ aggregation, thereby suppressing its adverse side effect.

## Introduction

The number of patients affected with Alzheimer’s disease (AD) is increasing^1^. AD is a slow progressing, neurodegenerative disorder, which exhibits the cardinal hallmarks of amyloid beta (Aβ) plaques accumulation and neurofibrillary tangles. In addition, the loss of synapses, atrophy of the cerebral cortex, neuroinflammation, and increase in free radicals in patients with AD is also reported^2^.

In the progression of AD, the conformation and misfolding of Aβ proteins causes them to clump together into small oligomers, protofibrils, and mature fibrils^3^. The soluble oligomers are structurally heterogeneous, which have been determined as the main toxic forms in AD^4^. The oligomer-induced toxicity of the cell is related to its ability to permeabilize cellular membranes and lipid bilayers^5^ to form distinct pores or single channels in the membranes^6^ and disturb intracellular calcium homeostasis, leading to oxidative stress including overproduction of reactive oxygen species (ROS), altered signaling pathways, mitochondrial dysfunction, and neuronal death^7^.

Currently, one of the most promising strategies for AD therapy is to decrease Aβ aggregation, especially Aβ(1-42) and/or destroy the oligomers and fibrils formed in the brain^8, 9^. Previous studies have proposed an effective inhibitor of Aβ, Quercetin, which blocks the formation of fibrils, including the inhibition of Aβ aggregation^10^, reduction in ROS production^11^, and alteration in the functions of immune and inflammatory cells^12^. Therefore, there are several targets such as inhibition of Aβ aggregation, and Aβ-induced oxidative stress, especially ROS, to improve therapeutic and preventive strategies for the development of disease-modifying drugs for AD.

Antioxidants are widely used to scavenge ROS^13, 14^. Although versatile antioxidants such as vitamin C and E, many phytochemicals, and synthetic drugs have been developed so far, none of them work effectively. One of the serious issues in the use of antioxidants is the dysfunction of normal redox reaction in healthy cells, including the electron transport chain due to the internalization of these low-molecular-weight (LMW) antioxidants because of their size. This undesired adverse side effect limits the effective dosage of these LMW antioxidants. Recently, we designed polymer antioxidants, where the antioxidant moieties are covalently bound to the amphiphilic block copolymer backbone. Due to the high molecular weight, the internalization of these polymer antioxidants is decreased in healthy cells and their mitochondria, which significantly reduce its adverse effects^15, 16^.

We designed the polymer poly(ethylene glycol)-b-poly[4-(2,2,6,6-tetramethylpiperidine-1-oxyl)aminomethylstyrene] (PEG-*b*-PMNT). The PEG segment is water-soluble part, while PMNT is the water-insoluble part. Thus, it forms a polymer micelle of several tens of nanometer size in transparent aqueous media (we abbreviate it as RNP^N^, as shown in Supplementary Figure 1). The 2,2,6,6-tetramethylpiperidine-1-oxyl (so called TEMPO) groups as a side chain of the PMNT segment is known to be a stable radical and does not react with each other. However, it catalytically reacts with ROS and is regarded as one of the strongest antioxidants^17^. Because the PMNT segment possesses repeating amino groups, it protonates in response to pH decrease and becomes hydrophilic to result in disintegration under acidic conditions^15, 18, 19^. After oral administration of RNP^N^ solution, it disintegrates in the stomach, and the disintegrated antioxidant polymer is absorbed in the bloodstream from the small intestine because the total molecular weight of the polymer was limited to around 7–20 kDa^19^. We have previously confirmed that our redox nanoparticle exhibits an anti-apoptotic effect on Aβ-induced cell death *in vitro* ^20^. We have also confirmed that oral administration of RNP^N^ showed an antioxidant effect on the brain of senescence-accelerated (SAMP8) mice and did not exhibit any detectable toxicity in the vital organs after continuous administration for 1 month^19^. The objective of this work was to confirm the effectiveness of RNP^N^ in transgenic AD mice model (Tg2576) for an extended time by ad libitum drinking of RNP^N^ for the prevention of Aβ accumulation in Tg2576 mice overexpressing a mutant form of amyloid precursor protein (APP).

## Results

### Delivery of redox polymer to the blood and brain by chronic ad libitum drinking of RNP^N^ solution

**Figure 1:**
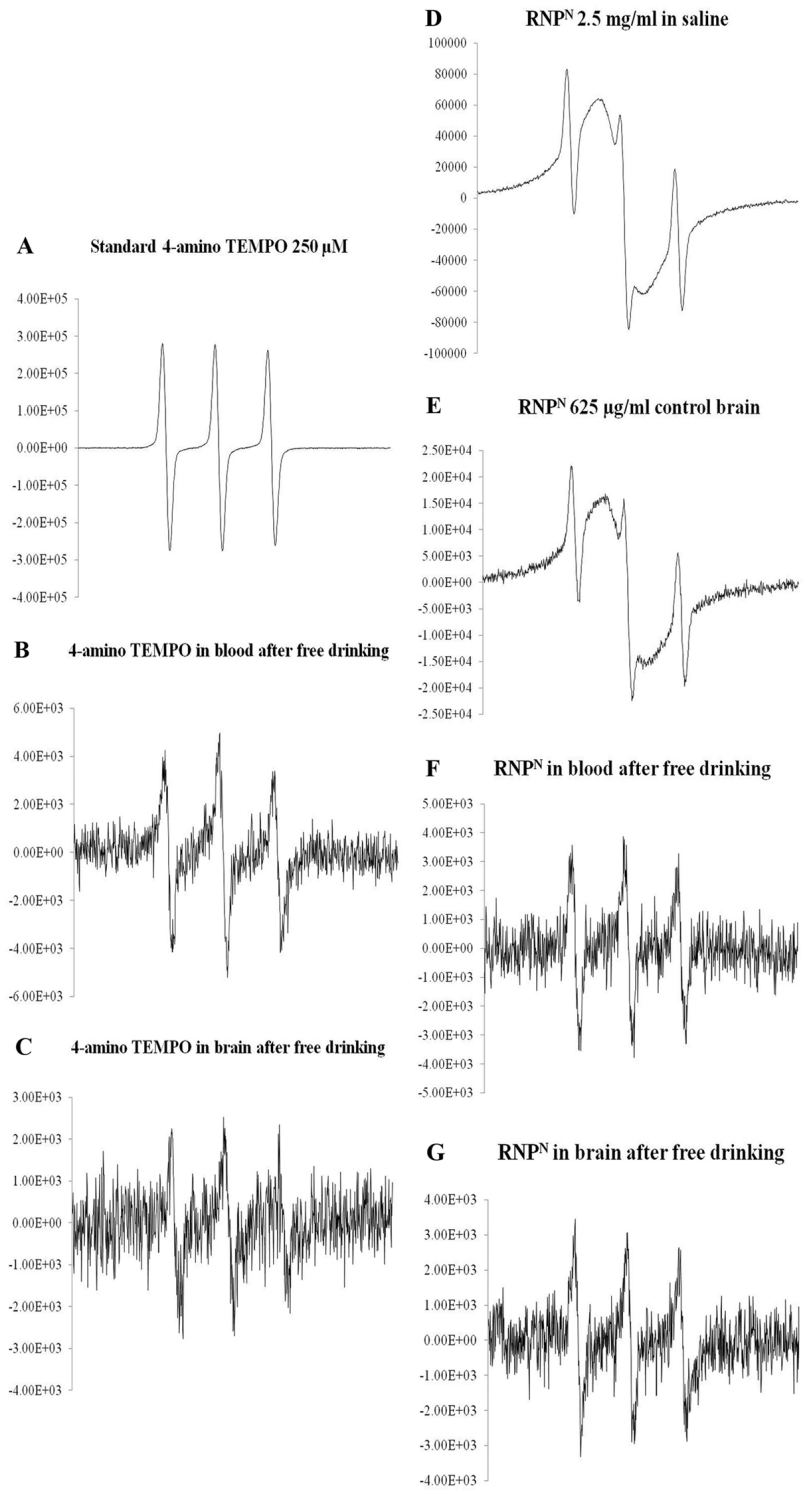
The electron spin resonance (ESR) spectra of 4-amino-TEMPO standard in saline (**A**), 4-amino-TEMPO in the blood after ad libitum drinking for 6 months (**B**) 4-amino-TEMPO in the brain after ad libitum drinking for 6 months (**C**) ESR spectra of RNP^N^ in saline (**D**), RNP^N^ in control brain (**E**), RNP^N^ in the blood after ad libitum drinking for 6 months (**F**), and RNP^N^ in brain after ad libitum drinking for 6 months (**G**).

To confirm the uptake of the redox polymer in the blood by oral administration, electron spin resonance (ESR) measurements of the blood and brain were carried out. The ESR signal of LMW TEMPO derivatives showed a triplet signal due to the coupling between the unpaired electron on oxygen and ^14^N nuclei (Fig. 1A). After ad libitum drinking of LMW TEMPO, the triplet signal was clearly observed in both the blood and brain samples as observed in the standard solution (Fig. 1B,C). We have previously reported that the ESR pattern of an aqueous solution of RNP^N^ does not appear as a typical triplet signal but as broadened spectra as shown in Fig. 1D. When RNP^N^ was mixed with untreated brain, and the homogenate was analyzed by ESR measurement, the broadened spectrum was observed, similar to RNP^N^ in saline solution (Fig. 1E). After ad libitum drinking of RNP^N^ solution, small but definite signal was observed in the homogenized brain samples, although the signal pattern changed from broad ESR spectra (Fig. 1E) to triplet signal in both the blood and brain samples (Fig. 1F and G), indicating the disintegration of RNP^N^ in the stomach to result in the uptake of redox polymer in the bloodstream and brain tissues. These results were consistent with the quantitative analysis of ESR spectra intensities as displayed in Supplementary Table S1.

### Effect of redox polymer on learning and memory deficit in transgenic mice expressing mutant APP

**Figure 2:**
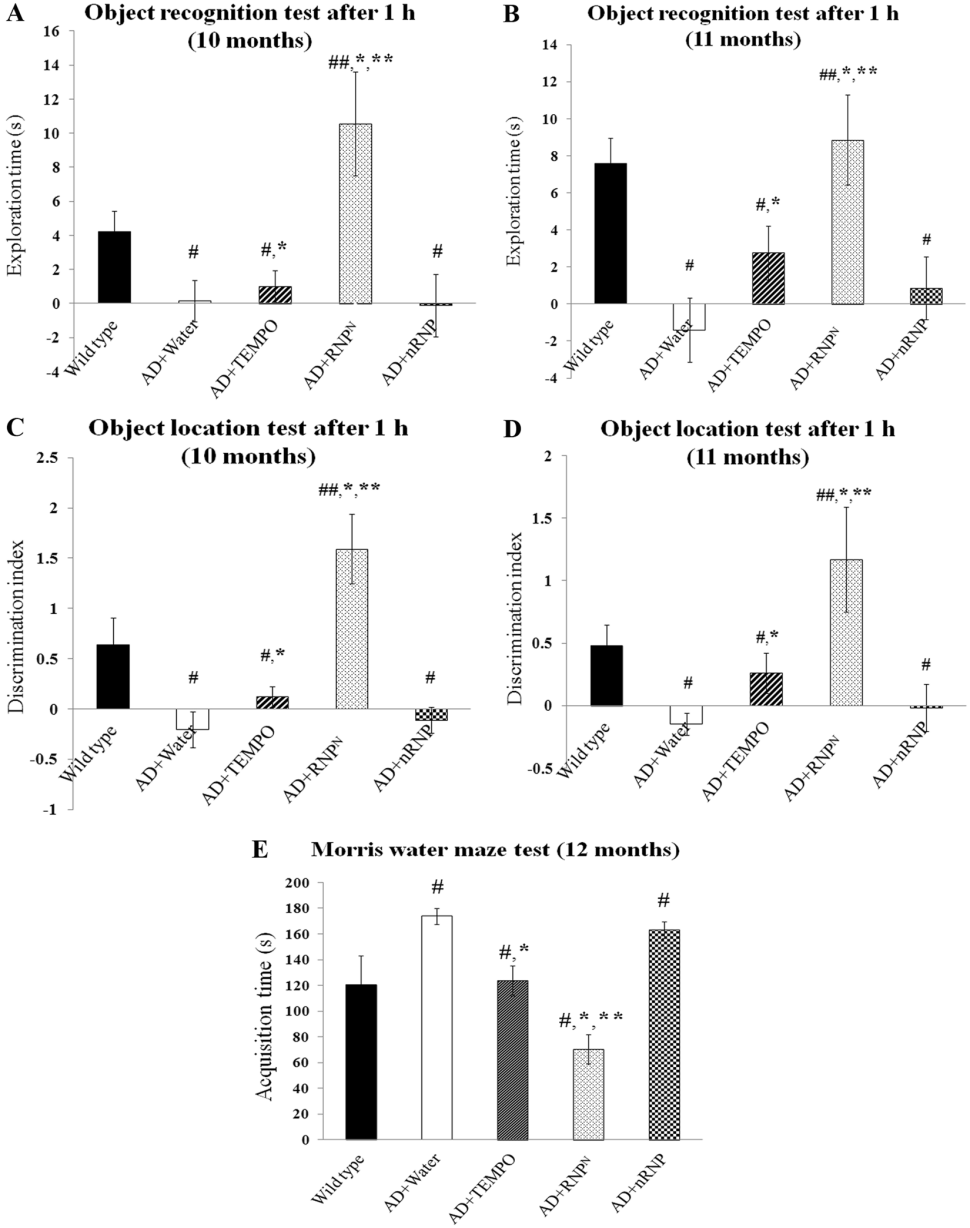
The chronic effect of oral ad libitum RNP^N^ treatment on object recognition test at mice age 10 (**A**) and 11 months (**B**), object location test at mice age 10 (**C**) and 11 months (**D**) after 1 h of administration and Morris water maze test at mice age 12 months (**E**). Data were represented as mean ± SEM, ^#^ vs. wild type mice, *p* < 0.05; ^##^ vs. AD mice treated with TEMPO, *p* < 0.05; * vs. AD mice treated with water, *p* < 0.05; ** vs. AD mice treated with nRNP, *p* < 0.05; n = 10 mice/group.

We analyzed the recovery of learning and memory functions in transgenic mice expressing APP in the brain as shown in Fig. 2. In the object recognition and location memory tests, the time period was lower for AD mice aged around 10–11 months, compared with the wild type group while in the groups treated with LMW TEMPO and nRNP, which is a polymer micelle without TEMPO moiety, the exploration time and discrimination index did not increase. In contrast, the exploration time and discrimination index for the RNP^N^-treated group significantly increased compared with other treated groups for both the object recognition and location tests (Fig. 2A–D). Furthermore, the effect of RNP^N^ on object recognition test after 24 h also displayed the same pattern as after 1 h of ad libitum drinking (Figure S4). The Morris water maze tests also showed a tendency similar to the object recognition tests; the RNP^N^-treatment significantly shortened the acquisition time compared with other groups (Fig. 2E). The aforementioned results confirmed that the RNP^N^-treatment significantly improved the learning and memory dysfunctions in AD transgenic mice (*p* < 0.05) when compare to the water-treated, LMW TEMPO-treated, and nRNP-treated groups. It is interesting to note that the LMW TEMPO-treated group did not exhibit a strong improvement effect on the learning and memory functions than those of RNP^N^-treated mice, although it was detected in the brain using ESR measurements.

### Suppression of oxidative stress in the brain by ad libitum drinking of RNP^N^ solution

**Figure 3:**
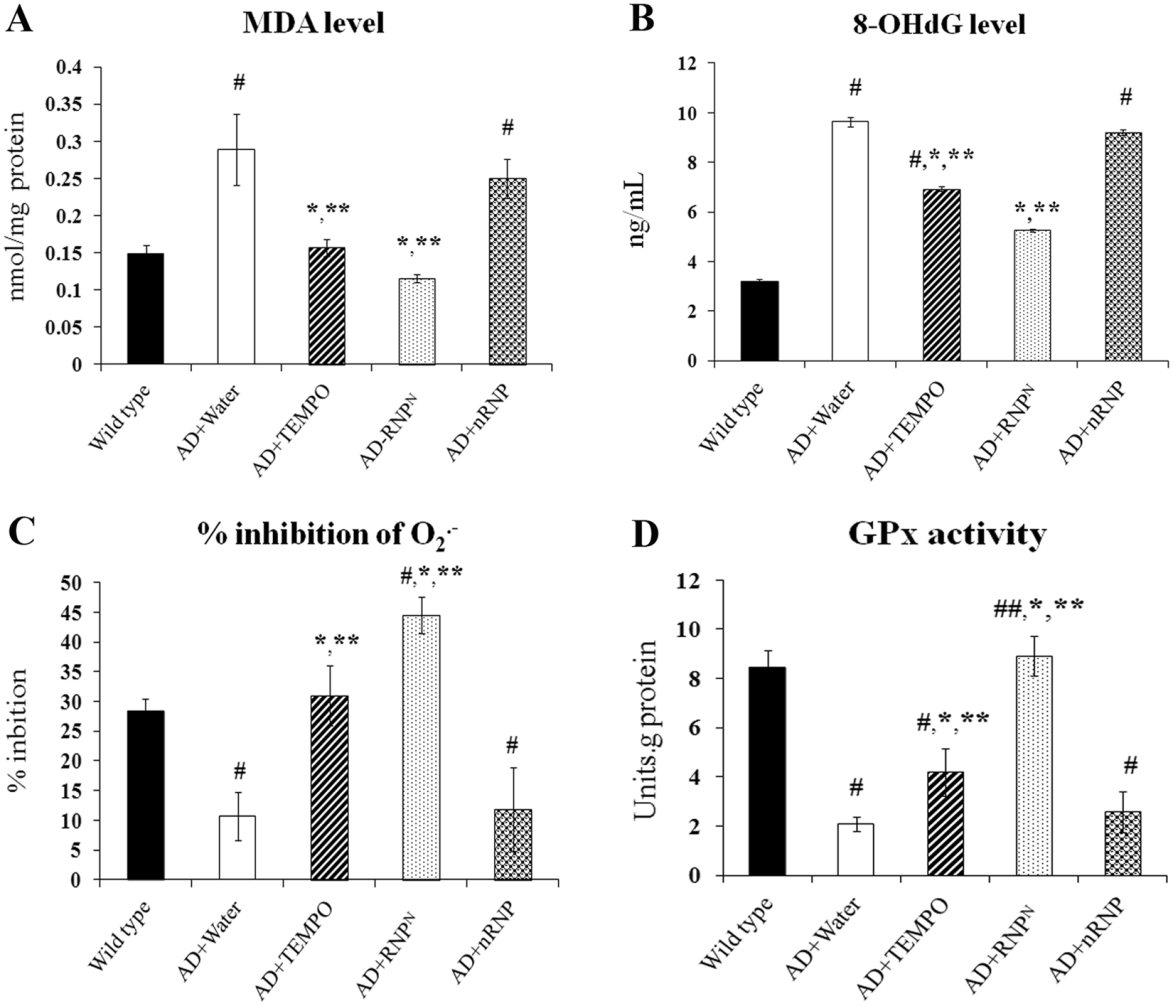
The oral ad libitum treatment with RNP^N^ improves oxidative stress parameters of MDA level (**A**), 8-OHdG level (**B**), % inhibition of O_2_^·−^ (**C**) and GPx activity (**D**). Data were represented as mean ± SEM, ^#^ vs. wild type mice, *p* < 0.05; ^##^ vs. AD mice treated with TEMPO, *p* < 0.05; * vs. AD mice treated with water, *p* < 0.05; ** vs. AD mice treated with nRNP, *p* < 0.05; n = 5 samples/group.

The lipid peroxide or malondialdehyde (MDA), 8-hydroxy-2′ -deoxyguanosine (8-OHdG) levels, the percent inhibition of superoxide anion (O_2_^·−^), and glutathione peroxidase (GPx) assays were used to determine the free radical scavenging capacity of RNP^N^ in comparison with non-treated, water-treated, TEMPO-treated, and nRNP-treated groups. As shown in Fig. 3, RNP^N^ treatment decreased MDA and 8-OHdG levels, while increasing percent inhibition of O_2_^·−^ and GPx activity when compared to the other treatment groups (*p* < 0.05), indicating the effectiveness of RNP^N^ treatment compared to LMW antioxidant treatment.

### Decreased Aβ(1-40), Aβ(1-42), and γ-secretase levels in the brain and plasma of transgenic mice treated with ad libitum drinking of RNP^N^ solution

**Figure 4:**
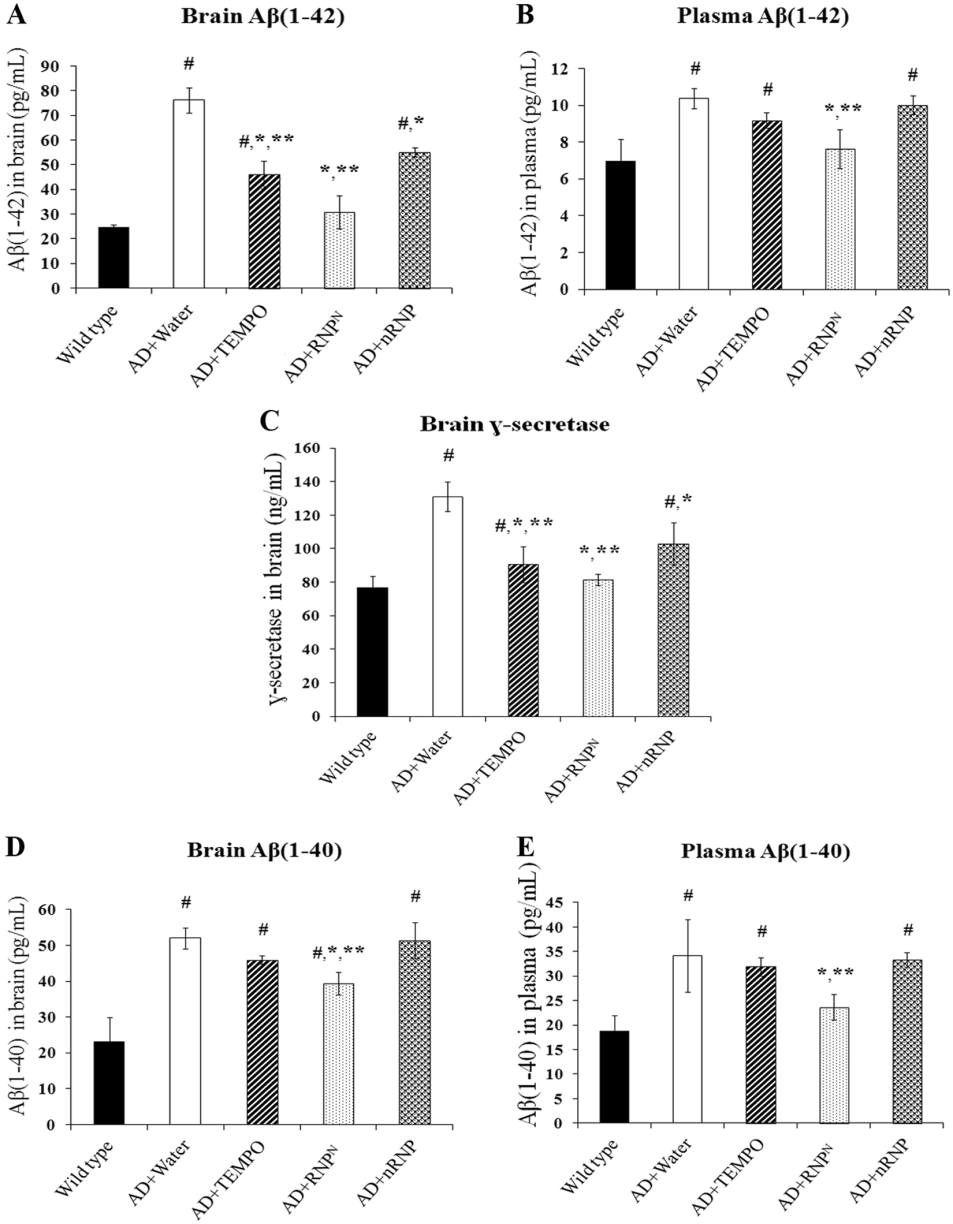
The effect of ad libitum drinking of RNP^N^ on Aβ(1-42) in the brain (**A**), the plasma (**B**), γ-secretase in the brain (**C**) and Aβ (1-40) in the brain (**D**), Aβ(1-40) in the plasma (**E**) of mice. Data were expressed as mean ± SEM, ^#^ vs. wild-type group, *p* < 0.05; * vs. AD mice treated with water, *p* < 0.05; ** vs. AD mice treated with nRNP, *p* < 0.05, n = 5 samples/group.

As mentioned above, the formation and aggregation of Aβ(1-42) is more toxic, widespread, and abundant than Aβ(1-40)^21^. In addition, γ-secretase is a protease with substrates that cleave APP and catalyze Aβ aggregation via the amyloidogenic pathway^22^. We observed that the levels of Aβ(1-40), (1-42), and γ-secretase in both the brain and plasma samples were significantly reduced after RNP^N^-treatment compared to AD mice treated with water, TEMPO, and nRNP (*p* < 0.05) as shown in Fig. 4.

### Reduction in amyloid plaque formation by chronic ad libitum drinking of RNP^N^ solution

**Figure 5:**
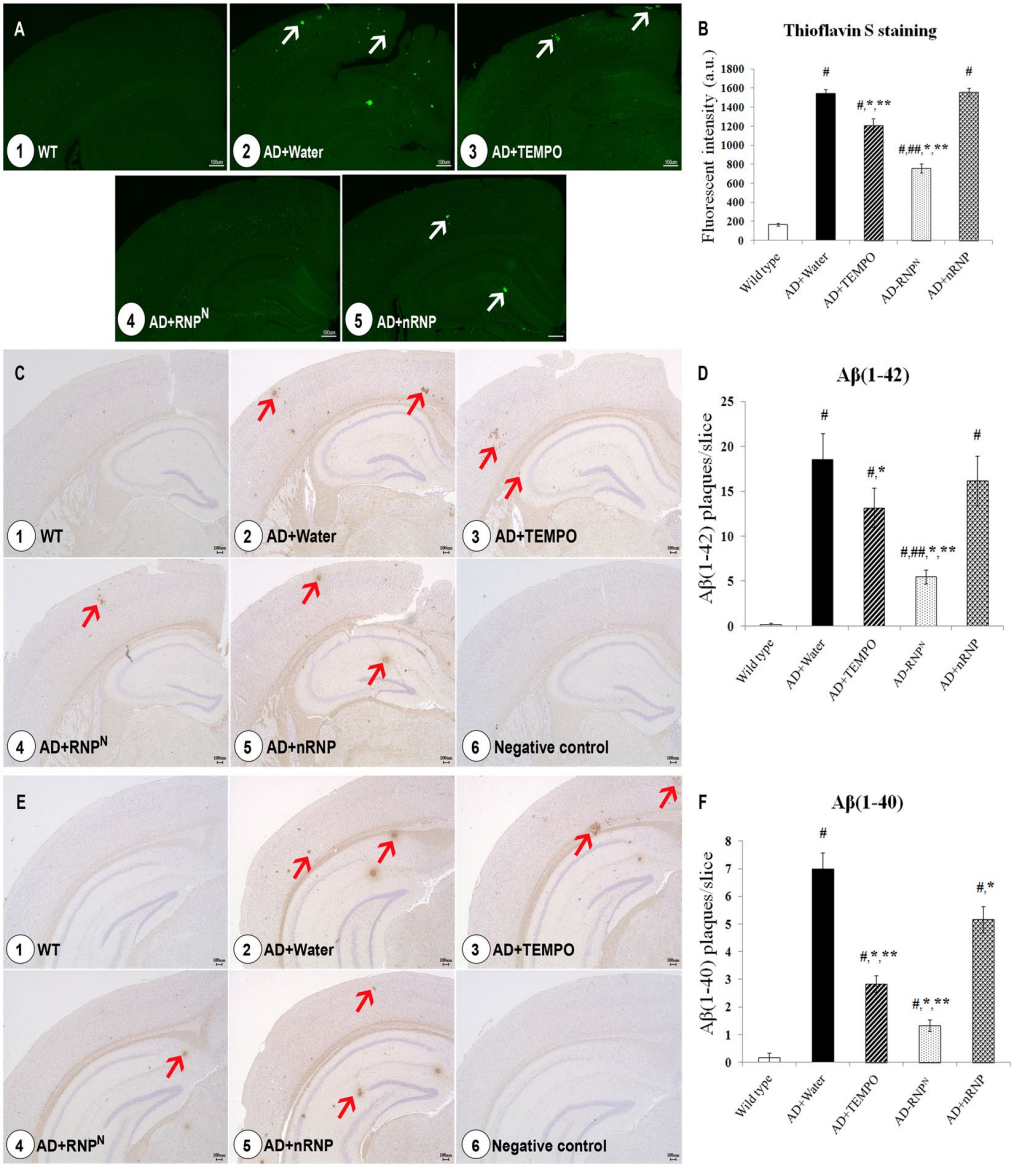
The effect of oral ad libitum drinking of RNP^N^ on Aβ-fibrils using thioflavin S staining (**A**), semi-quantitative analysis of Aβ-fibril (**B**), immunohistochemistry staining of Aβ(1-42) (**C**), semi-quantitative analysis of Aβ(1-42) (**D**), immunohistochemistry staining of Aβ(1-40) (**E**), and semi-quantitative analysis of Aβ(1-40) (**F**) of the cerebral cortex of mice brain; 1. wild type group; 2. AD mice group treated with water; 3. AD mice group treated with TEMPO; 4. AD mice group treated with RNP^N^; 5. AD mice group treated with nRNP; 6. negative control group. Data were expressed as mean ± SEM, ^#^ vs. wild-type group, *p* < 0.05; ^##^ vs. AD mice treated with TEMPO, *p* < 0.05; * vs. AD mice treated with water, *p* < 0.05; ** vs. AD mice treated with nRNP, *p* < 0.05, n = 5 sample/group. White arrow indicates amyloid fibril and red arrow indicates amyloid plaque and scale bar = 100 μm.

To confirm amyloid plaque formation and/or aggregation in mice brain, thioflavin S and immunohistochemistry staining of Aβ(1-40) and Aβ(1-42) were carried out. From the representative photomicrographs of thioflavin S staining in the cerebral cortex, as shown in Fig. 5A, no remarkable Aβ fibrils was observed in wild-type mice (Fig. 5A-1), while transgenic mice in the water-treated group (Fig. 5A-2) showed remarkable Aβ fibrils in the cortex area. Although the LMW TEMPO treatment attenuated the number Aβ fibrils to some extent, AD mice treated with RNP^N^ showed much higher attenuation efficiency (Fig. 5A-4) (p < 0.05, Figs 5A-3,5), which was confirmed by the quantitative fluorescent intensity in Fig. 5B.

The immunostaining of Aβ(1-40) and Aβ(1-42) revealed no immunoreactive cells in the cerebral cortex of wild-type mice (Fig. 5C-1, E-1). On the contrary, in AD transgenic mice that were treated with water, numerous immunoreactive Aβ(1-40) and Aβ(1-42) positive cells were observed (Fig. 5C-2, E-2), similar to that after treatment with nRNP (Fig. 5C-5, E-5). The RNP^N^-treated AD mice group showed significant reduction in Aβ(1-40) and Aβ(1-42) positive cells (Fig. 5C-4, E-4). This reduction was higher than that in the LMW TEMPO group (Fig. 5C-3, E-3), which are quantitatively confirmed as shown in Fig. 5D,F. For quantitative evaluation of number of plaques as shown in Fig. 5D,F, we employed randomly captured 5 slices per group under magnification of 20X (See Figure S5).

## Discussion

We have designed an antioxidative treatment in a transgenic AD model mice (Tg2576) using our novel amphiphilic polymer antioxidant, which self-assembles in aqueous media. Since the size of RNP^N^ was approximately 30-40 nm, we also confirmed that the volume of ad libitum drinking of RNP^N^ solution was the same as in water, TEMPO, and nRNP-treated mice (data not shown). The triplet signals were observed in both the blood and brain samples of LMW TEMPO-administered group (Fig. 1B and C), suggesting that the blood brain barrier of these Tg2576 mice might be weakened at tight junctions between the endothelial cells due to the significant Aβ expression^23^. Immediately, after preparation via the dialysis method, the ESR signal of RNP^N^ became broader, because of the restricted mobility of the nitroxide radicals in the solid state, which proves that the confinement of nitroxide radicals in the nanoparticle core (Fig. 1D). The ESR signals of RNP^N^ changed its pattern from broad ESR spectra to triplet in the blood of mice with ad libitum drinking (Fig. 1F) due to its disintegration in the stomach followed by absorption in the blood stream via mesentery in the small intestine. Because the redox polymer possesses cationic charge, it was previously confirmed to interact with serum proteins in the blood stream and circulate for an extended period^19^. By the ad libitum drinking of RNP^N^ solution, the redox polymer continuously circulates in the bloodstream and gains access to the brain vessel walls continuously. This repeated access of the redox polymer improved the uptake in the brain as shown in Fig. 1G. The results of ESR signal intensity (a.u.) data (Supplementary Table S1) exhibited that the brain of mice treated with RNP^N^ (3.04 × 10^3^ a.u.) has significantly higher ESR signal intensity than that of the LMW TEMPO-treated group (1.94 × 10^3^ a.u.), which is probably due to the rapid elimination of LMW TEMPO.

Transgenic Tg2576 mice overexpressing human amyloid precursor protein (hAPP) are widely used as an AD mouse model to evaluate the treatment effects on Aβ pathology. Previous reports on the onset of cognitive deficits in Tg2576 have determined that abnormalities appear as early as 3 months and as late as 12 months^24^. In this study, the aged Tg2576 mice (approximate 7–12 months) were used for the evaluation of the learning and memory of AD mice; Morris water maze, novel object recognition and object location memory tests^25–28^. The transgenic mice we used in this study showed statistically significant reduction of the duration of both object recognition and location tests and elongation of acquisition time in Morris water maze test than that of wild-type mice, which is in accordance with the previous study where Tg2576 mice displayed impaired spontaneous alternation of Y-maze test at both 3 and 10 months of age, as well as advanced impairment in the acquisition time of Morris water maze test in 9–10 months of AD mice^29^. We have previously reported that oral RNP^N^ treatment recovered both cognition and memory levels in 17-week-old SAMP8 mice^19^. Here, RNP^N^-treated group extended the time in the object recognition and location tests and decreased the acquisition time in Morris water maze test, indicating the effective attenuation of the learning and memory deficits in AD transgenic mice (Tg2576) (Fig. 2). Taking together these results and our previous data on SAMP8, it suggests that our antioxidative nanoparticle treatment is robust strategy for AD therapy.

Oxidative stress has been implicated in Aβ accumulation and progression via mitochondrial dysfunction, which caused by the generation of ROS^30, 31^ and reduction in the level of detoxifying enzymes including superoxide dismutase (SOD), GPx and catalase (CAT) in the early stages of the AD^32, 33^. ROS destroys the polyunsaturated fatty acids of cellular membranes to generate lipid peroxidation products such as MDA, which may serve as an indicator of the level of oxidative damage^32, 34^, and induce neuronal deterioration^35, 36^. In addition, 8-OHdG has been determined as a pivotal biomarker of oxidative DNA damage^37^. Additionally, the toxic effect of Aβ peptide in neuronal cells has been proposed via the interaction between the peptide and Cu^2+^ and Fe^3+^ ions because Aβ is a metalloprotein that displays high-affinity binding to these ions, leading to amyloid plaque formation. Aβ catalyzes the reduction of Cu^2+^ to Cu^+^ and Fe^3+^ to Fe^2+^, that produce hydrogen peroxide (H_2_O_2_)^38–40^. Furthermore, in AD transgenic mouse models of mutants of APP elevated production of H_2_O_2_ and nitric oxide increases protein and lipid peroxidation. These were associated with age-related Aβ accumulation, and Aβ further enhances oxidative stress^41^. As expected, the observed neuroprotection by RNP^N^ was due to its antioxidant properties through enhancement of the activity of GPx and percent inhibition of O_2_^·−^, while reducing MDA and 8-OHdG levels, compared to other AD groups (*p* < 0.05) as shown in Fig. 3. This was consistent with our previous result, where we showed that RNP^N^ eliminates superoxide anion and hydroxyl radicals, which cause lipid peroxidation and protein and DNA oxidation^19, 20^.

Since RNP^N^ possesses covalently conjugated TEMPO molecules, which scavenge superoxide radicals^42^ it directly reacts with both carbon-centered and peroxy radicals^43^ preventing the reduction of hydrogen peroxide to hydroxyl radical^44^. Hence, RNP^N^ might attenuate the formation of hydroxyl radical and acts similarly to SOD. The LMW TEMPO compounds easily permeate the cell membrane and functions as an intracellular scavenger of O_2_^·−^ and other radicals^45, 46^, which is in sharp contrast to our redox polymer because of no internalization in healthy cells due to the high molecular weight^16^. Although our redox polymer, PEG-b-PMNT was not internalized in healthy cells, it was effective in APPsw/Tg2576 AD mice to attenuate oxidative damage and induced the recovery of the activities of the radical scavenging enzymes of GPx. The suppression of cell internalization tendency of the redox polymer is one of the most important factors to suppress severe adverse side effects in healthy cells and tissues unlike the LMW TEMPO^16^. Recently, we have been investigating the effect and potential toxicity of RNPs (pH-sensitive RNP^N^ and pH-insensitive RNP^O^), TEMPOL and nRNP to several animals. For example, when zebrafish larvae were exposed to 10–30 mM of LMW TEMPOL, all were dead after 12 h, whereas no larva death was observed after exposure to RNPs at the same high concentrations. By staining mitochondria from the larvae, we found that LMW TEMPOL significantly induced mitochondrial dysfunction. In contrast, RNPs did not cause any significant reduction in the mitochondrial function of zebrafish larvae^16^. Oral administration of RNP^O^ to healthy mice did not cause any damage to intestinal microflora^47^. In addition, long-term oral administration of RNP^O^ to xenograft-tumor-bearing mice coupled with anti-cancer drug did not cause any adverse effect but reduced diarrhea and other undesired phenomena^48–50^. Oral administration of RNP^N^ to transgenic non-alcoholic steatohepatitis model mice suppressed liver fibrosis but cause no adverse effect^51^. On the basis of our alternative investigations, we have concluded that RNPs do not cause strong toxic and adverse effects. In contrast to conventional antioxidants that can be internalized into healthy cells, which destroy its normal redox reactions (e.g., electron transport chain), the size of RNPs prevents its cellular internalization.

ROS are implicated in the formation of senile plaques in the brains of patients with AD, which may result in neuronal death^30^. As the result displayed that scavenging ROS by orally administered RNP^N^ significantly attenuates the neuronal loss in both the cerebral cortex and hippocampus when compared to other AD-treated groups (Supplementary Figures S2-3, *p* < 0.05).

The development of γ-secretase inhibitors has been explored as drugs for AD^52^. Our *in vivo* result shows that RNP^N^-treatment significantly reduced γ-secretase levels, followed by a decrease in Aβ-level compared to AD mice treated with water, TEMPO, and nRNP respectively (*p* < 0.05), as shown in Fig. 4, which is in accordance with the previous *in vitro* and knockdown experiments.

Finally, we confirmed amyloid plaque formation and/or aggregation via thioflavin S and immunohistochemistry staining of Aβ(1-40) and Aβ(1-42). The results shown in Fig. 5 display that chronic oral administration of RNP^N^ significantly reduced Aβ plaques and/or fibril aggregation when compared to other AD treatment groups (*p* < 0.05). It has been reported that the accumulation of Aβ is through the interaction of soluble Aβ with metal ions, mainly Zn^2+^, Cu^2+^, and Fe^3+^ ^38^. The prevention of soluble Aβ formation by RNP^N^ treatment inhibits Aβ aggregation.

## Conclusion

Here, we confirmed that after ad libitum drinking of our pH-sensitive redox nanoparticle, RNP^N^, which is a self-assembling polymer antioxidant, RNP^N^ is internalized in the brain and eliminates the increased oxidative stress by scavenging ROS and attenuates cognitive deficits. The increased levels of Aβ and γ-secretase and radical-scavenging activity were decreased after RNP^N^ treatments. Finally, the number of the thioflavin S-stained neurons was significantly higher in the RNP^N^-treated group than in other groups. On the basis of these obtained results in addition to our previous results on the senescence-accelerated mice-treated with RNP^N^, we suggest that RNP^N^ may be a promising candidate for the treatment of brain disorders including AD therapy.

## Methods

### Drugs and reagents

Amino-2,2,6,6-tetramethylpiperidine-N-oxyl (TEMPO), 4-hydroxy-2,2,6,6-tetramethylpiperidine-1-oxyl (TEMPOL) were purchased from Sigma-Aldrich (MO, USA), Mouse Aβ(1-42), Aβ(1-40) and γ-secretase ELISA kits were bought from MyBioSource, Inc. (San Diego, USA), Oxidative DNA damage ELISA kit was purchased from Cell Biolab, Inc. (San Diego, USA), Liquid 3,3′-diaminobenzidine (DAB) substrate kit was bought from Life technologies Corp. (Waltham, USA) and primary anti-Aβ(1-42), Aβ(1-40) and secondary antibodies were purchased from Abcam Plc. (Tokyo, Japan). All other chemicals in this study, which are analytical-reagent grade, were purchased locally from Wako Pure Chemical Industries Ltd. Japan.

### Animals

Female APPSWE/hemi-rd1 Tg2576 and wild-type mice were used in this study (CLEA, Japan, Inc., Tokyo, Japan). They were housed in the experimental animal center of the University of Tsukuba under controlled temperature (23 ± 1 °C), humidity (50 ± 5%), and lighting (12 h light/dark cycles). The animals had unrestricted access to food and water. All the experiments were carried out in accordance with the guidelines for animal care and use of Faculty of Medicine, Tsukuba University and were approved by the animal ethics committee of the Institutional Animal Experiment Committee at the University of Tsukuba (Protocol number 13-407) and in accordance with the Regulation for Animal Experiments in our University and the Fundamental Guidelines for Proper Conduct of Animal Experiments and Related Activities in Academic Research Institutions under the jurisdiction of the Ministry of Education, Culture, Sports, Science, and Technology.

### Biodistribution of RNP^N^ in the blood and brain after ad libitum drinking

Tg2576 mice age approximately 12 months old were anesthetized via an intraperitoneal injection of sodium pentobarbital (50 mg/kg) after 6-month ad libitum drinking of RNP^N^ solution (5 mg/mL/day) and LMW TEMPO (0.6 mg/mL/day). The blood and brain tissue were collected immediately after perfusion. Whole blood samples were subjected immediately to ESR measurement to quantify the drug levels. The brain tissue was immediately placed on ice and homogenized.

The ESR signals from the blood were recorded at room temperature using a Bruker EMX-T ESR spectrometer operating at 9.8 GHz with a 100 kHz magnetic field modulation. Signals were collected with the following parameters: center field, 5000 G; sweep width, 7000 G; microwave power, 100.2 mW; receiver gain, 1 × 10^3^; time constant, 81.92 ms; and conversion time, 160 ms and sweep time, 163.84 s. The blood samples were corrected at predetermined time points and subjected to the ESR.

The total amount of drug (nitroxide radicals + hydroxyamines) in the brain was estimated from X-band ESR spectrometer (JES-TE25X; JEOL, Tokyo, Japan) at room temperature after the oxidation of hydroxylamine by K_3_[Fe(CN)_6_], which was prepared at 200 mM as a stock solution. The ESR measurements were carried out under the following conditions: frequency, 9.41 GHz; power, 8.00 mW; field, 333.8 ± 5 mT; sweep time, 1.0 min; modulation, 0.1 mT; time constant, 0.1 s.

### Voluntary test fluids consumption

Fifty milliliters of drinking fluids were provided in 75 mL drinking bottles equipped with stainless steel spouts, placed on cage covers. During the first 5 days, mice were given water as their only drinking fluid from bottles replacement water tap system to habituate them. For the next 6 months (at 7–12 months of age), all mice were allowed drinking bottles containing RNP^N^ (5 mg/mL), nRNP (5 mg/mL), LMW TEMPO (0.6 mg/mL), and water in each group. The consumption of the fluids and the body weights was recorded once a week, and the bottles were filled with fresh solutions.

### Experimental design

We used 7 month-old female APPSWE/hemi-rd1 Tg2576 and their non-transgenic littermates or wild-type (WT) mice (40 Tg2576 mice and 10 WT mice; weight, 25 ± 2.0 g and 30 ± 2.0 g, respectively) in this study. All Tg2576 mice were randomly assigned to various groups for RNP^N^ (5 mg/mouse/day) or blank micelles (5 mg/mL/day) or LMW TEMPO (0.6 mg/mL/day) or vehicle (water) by unrestricted access from drinking bottles every day for a 6-month period. In the fourth and fifth month after ad libitum drinking, the animals were tested in object recognition and object location tests. In the last month of the experiment, we also tested the mice in the Morris water maze test. After 6 months of the experiment, 50 mice were sacrificed, and half of the brain tissues were used for the assays for soluble Aβ(1-40) and Aβ(1-42), γ-secretase activity, ROS production (MDA and DNA oxidation), ROS levels, scavenging enzyme activity (GPx activity), and the other halves were used for Aβ immunohistochemical, thioflavin S, and cresyl violet staining.

### Plasma and brain collection

After their behavioral tests were finished, the mice were anesthetized with sodium pentobarbital (50 mg/kg i.p.); blood samples were kept to separate plasma, and brains were quickly isolated. The tissues were prepared for biochemical, histological and immunohistochemical examinations and stored at −80 °C until determination.

### Antioxidant enzyme assay

The glutathione peroxidase (GPx) activity was determined by the previous method of Hussain *et al*.^53^ based on that the activity was measured indirectly by a coupled reaction with glutathione reductase. Oxidized glutathione, produced upon reduction of hydrogen peroxide by glutathione peroxidase, was recycled to its reduced state by glutathione reductase and NADPH. The oxidation of NADPH to NADP^+^ was accompanied by a decrease in absorbance at 340 nm. The rate of decrease in the A340 nm was directly proportional to the glutathione peroxidase activity. In the final 1 mL of the system mixture contained 48 mM sodium phosphate, 0.38 mM EDTA, 0.12 mN β-NADPH, 0.95 mM sodium azide, 3.2 units of glutathione reductase, 1 mM glutathione (GSH), 0.02 mM DL-dithiothreitol, 0.0007% H_2_O_2_, and the standard enzyme glutathione peroxidase solution or a homogenate brain sample. The glutathione peroxidase solution was used as a standard enzyme activity. The standard curve was plotted as the rate of A340 nm per minute against the GPx activity. One unit activity was defined as the amount of enzyme necessary to catalase the oxidation by H_2_O_2_ of 1 µmole of GSH to GSSG per minute at pH 7 at 25 °C. The data were reported in units of GPx per mg protein.

### Reactive oxygen species (ROS) products assays

Lipid peroxidation (LPO) was measured by determining malonyldialdehyde (MDA) which have been used as an indicator of lipid peroxidation according to Ohkawa *et al*.^54^, were measured by using a commercial assay kit (BIOMOL International, USA). Each sample was homogenized (Potter-Elvehjem) in a 10-fold volume of ice-cold 20 mM pH 7.4 of PBS containing 0.5 mM butylated hydroxytoluene to prevent sample oxidation. The homogenized sample was centrifuged at 3,000 *g* at 4 °C for 10 min, and a 200 µL aliquot of the supernatant was used to measure MDA plus HAE levels according to the instructions of the manufacturer. Values were standardized to micrograms of protein.

Each sample was homogenized (Potter-Elvehjem) in a 10-fold volume of ice-cold 20 mM pH 7.4 of PBS containing 1% streptomycin sulfate and incubated for 30 min at room temperature. The nucleic acid precipitates were removed by centrifuging at 6,000 *g* for 10 min at 4 °C to avoid erroneous contribution to a higher estimation of the carbonyl content from nucleic acid in the cells. The supernatant was used to measure protein levels according to the instructions of the manufacturer. The obtained values were standardized to milligrams of protein. Deoxyguanosine (dG) is one of the constituents of DNA and when it is oxidized, it is altered into 8-hydroxy-2′-deoxyguanosine (8-OHdG). 8-OHdG is useful as a general DNA oxidation marker in the body. A commercial assay kit (Cell Biolab, Inc., San Diego,USA) was used in this measurement. Tissue homogenate and supernatant were used to measure 8-OHdG levels according to the instructions of the manufacturer. The obtained values were standardized to milligrams of protein.

### Brain oxidative DNA damage determined by ELISA

The remained supernatant of mice’ brains were also analyzed of 8-hydroxydeoxyguanosine (8-OHdG) which is the common marker of DNA oxidative stress according to the protocol of product manual (Cell Biolab, Inc., San Diego, USA). First, prepared 8-OHdG coated plate, overnight at 4 °C. Washed, filled assay diluent to each well and incubated for 1 h at room temperature (RT). Removed the solution, added 50 µL of sample to the coated wells following with anti-8-OHdG antibody and incubated on an orbital shaker for 1 h at RT. Washed properly, filled 100 µL of secondary antibody. Added substrate solution and inhibit reaction by stop solution. Eventually, read the absorbance at 450 nm on a spectrophotometer to compare to the standard curve of 8-OHdG.

### Assay of superoxide anion level

The reaction mixture consisted of 10 mM phosphate buffer (pH 7.4) containing 0.1 mM xanthine, 0.1 mM EDTA, 0.1 mM nitroblue tetrazolium, and 0.1 unit xanthine oxidase (XO) at a final volume of 1 mL. The formation rate of formazan produced was determined from the slope of the absorbance curve during the initial 2 min of the reaction at 560 nm. In order to analyze the anti-oxidation activity, each sample of different groups was added to the reaction mixture. The change of absorbance was compared with that of the control in the same time reaction, and anti-oxidation activity was calculated according to the following Equation (1)

1 $$\mathrm{Anti}\mbox{-}\mathrm{oxidation}\,{\rm{activity}}\,( \% )=\mathrm{100}\times ({\rm{A}}-{\rm{B}})/{\rm{A}}$$

where A and B are the rate of formazan formation in the absence and presence of sample, respectively.

### Measurement soluble Aβ(1-40), soluble Aβ(1-42) and γ-secretase in brain tissue and plasma

The brain tissue from one brain hemisphere of each mouse was homogenized in PBS, pH 7.4 and centrifuged at 1500 g for 15 min. The anticoagulated blood was drawn and spinned at 1000 g for 10 min. The supernatants of both types of sample were collected and protein quantification was performed using the bicinchoninic acid (BCA) assay (Bio-Rad Laboratories, Hercules, CA). Samples were analyzed for soluble Aβ(1-40), Aβ(1-42) or γ-secretase (BioSource International, Inc., Camarillo, CA) according to the manufacturer’s instructions. Briefly, added 50–100 µL of samples to each well of pre-coated microtiter plate, mixed with 5–10 µL of balance solution, filled 50–100 µL of conjugate solution to each specimen and then incubated for 1 h at 37 °C on automated shaker. Washed properly, added substrate A and B respectively and then incubated for 10–15 min at 37 °C. Finally, the reaction was stopped by terminate solution and determine the optical density at 450 nm immediately to compare with the typical standard curve of mouse Aβ(1-40) or Aβ(1-42) or γ-secretase respectively.

### Histopathology

Brain was isolated for immunohistopathology. The corrected tissues were fixed in 10% formalin solution. Paraffin blocks were prepared after completing the tissue processing in different grades of alcohol and xylene. Brain sections (5 µm) were prepared from paraffin blocks using microtome, stained with 0.5% cresyl violet according to Paxions and Chorles^55^. Images were taken using OLYMPUS camera connected to the microscope to examine gross cellular damage and neuronal density determination using Image J^TM^ (NIH, MD, USA) software^56^.

### Aβ plaque staining and quantification

The brain sections of 5 µm were also studied of immunohistochemistry analysis for Aβ(1-40) and Aβ(1-42) respectively. Pre-heated slices with microwave in acetic solution, cool at RT, blocked with methanol and hydrogen peroxide solution for 30 min. Washed properly, prepared moist chamber, blocked with BSA and added primary anti-Aβ(1-40) and Aβ(1-42) respectively (1:100, 1:100 and 1:50) of each slices for overnight at RT. Washed again and incubated with secondary antibody conjugated-horseradish peroxidase (HRP) for 45 min. Developed color by using DAB kit and slices were represented as brown positive staining cells, compared to WT group and used without primary antibody as negative control group under light microscope (Olympus model BZ-X710 Keyence, Tokyo, Japan).

### Thioflavin S staining and semi-quantification

This method is suitable for staining of Aβ fibrils following Schmidt *et al*.^57^. After tissue processing, sections were immersed into 0.25% potassium permanganate solution for 20 min, bleaching solution for 2 min, blocking solution for 20 min, 0.25% acetic acid for 5 s, dropped thioflavin S staining solution on slices for 3–5 min, washed by 50% ethanol and following distilled water. Mounted with glycerin and observed under fluorescent microscope (Olympus model BZ-X710 Keyence, Tokyo, Japan).

### The behavioural tests

In this experiment using 3 behavioural tests for measuring cognition status. The Morris water maze test was selected as a method for the evaluation of the spatial learning and memory according to the Morris’s method^58^. A circular water tank (120 cm in diameter and 50 cm in height) was filled with water to a depth of 30 cm inside the tank; an escape platform (11 cm in diameter) was placed, with the top of 1 cm below the water surface. The platform was in the middle of the target quadrant, and its position remained fixed during the experiment. Above the tank, a white floor-to-ceiling cloth curtain was drawn around the pool, and four kinds of black cardboard (circle, triangular, rhombus and square) were hung equidistantly on the interior of the curtain serving as spatial cues. Each mouse had daily sessions of one trial for 5 consecutive days. When they succeeded, mice were allowed to stay on the platform for 30 s. When the mice failed to find the platform within 60 s, they were assisted by the experimenter and allowed to stay the platform for the same time. Probe trials were performed after the last training session at 6 months after free drinking substance.

The object location test or spatial novelty was conducted according to Barker *et al*. with some modification^59^. One day before object location test, all mice were exposed for 30 min to the empty test box for habituation. The object location tests consisted of 2 trials which are sample phase (T1) and test phase (T2) with a 30 min interval between the 2 trials. In sample phase, mice were exposed to object O1 and O2, which were placed in the far corner of the area. The animal was allowed to explore both objects during a sample phase for 3 min and the amount of exploration of each object was recorded. After a delay of 30 min, the test phase was started. In the test phase, object O3 was placed in the same position as object O1 in the sample test while object O4 was placed in the corner adjacent to the original position of O2, so that object O3 and O4 were in diagonal corners. Thus, both objects in the test phase were equally familiar, but one was in a new location. The position of the moved object was counterbalanced between mice. All measures experiments were made with the experimenter blind to the treatment status of each animal. The basic measure was the total time spent by mice exploring each object during T1 and T2 trials. Exploratory behavior was defined as the animal directing the nose toward the object at a distance of <2 cm. Looking around while sitting or resting against the object was not considered as exploration. On object location task the amount of time exploring each object (object in the new location versus object in familiar position) is reported as an object discrimination ratio and calculated using the following Equation (2)

2 $$\begin{array}{c}({\rm{E}}{\rm{x}}{\rm{p}}{\rm{l}}{\rm{o}}{\rm{r}}{\rm{a}}{\rm{t}}{\rm{i}}{\rm{o}}{\rm{n}}\,{\rm{t}}{\rm{i}}{\rm{m}}{\rm{e}}\,{\rm{o}}{\rm{f}}\,{\rm{o}}{\rm{b}}{\rm{j}}{\rm{e}}{\rm{c}}{\rm{t}}\,{\rm{i}}{\rm{n}}\,{\rm{t}}{\rm{h}}{\rm{e}}\,{\rm{n}}{\rm{e}}{\rm{w}}\,{\rm{l}}{\rm{o}}{\rm{c}}{\rm{a}}{\rm{t}}{\rm{i}}{\rm{o}}{\rm{n}}-{\rm{E}}{\rm{x}}{\rm{p}}{\rm{l}}{\rm{o}}{\rm{r}}{\rm{a}}{\rm{t}}{\rm{i}}{\rm{o}}{\rm{n}}\,{\rm{t}}{\rm{i}}{\rm{m}}{\rm{e}}\,{\rm{o}}{\rm{f}}\,{\rm{o}}{\rm{b}}{\rm{j}}{\rm{e}}{\rm{c}}{\rm{t}}\,{\rm{i}}{\rm{n}}\\ \quad {\rm{f}}{\rm{a}}{\rm{m}}{\rm{i}}{\rm{l}}{\rm{i}}{\rm{a}}{\rm{r}}\,{\rm{l}}{\rm{o}}{\rm{c}}{\rm{a}}{\rm{t}}{\rm{i}}{\rm{o}}{\rm{n}})/{\rm{T}}{\rm{o}}{\rm{t}}{\rm{a}}{\rm{l}}\,{\rm{e}}{\rm{x}}{\rm{p}}{\rm{l}}{\rm{o}}{\rm{r}}{\rm{a}}{\rm{t}}{\rm{i}}{\rm{o}}{\rm{n}}\,{\rm{t}}{\rm{i}}{\rm{m}}{\rm{e}}\,{\rm{o}}{\rm{f}}\,{\rm{b}}{\rm{o}}{\rm{t}}{\rm{h}}\,{\rm{o}}{\rm{b}}{\rm{j}}{\rm{e}}{\rm{c}}{\rm{t}}{\rm{s}}\end{array}$$

The object recognition task was performed in a circle open-field apparatus (60 cm in diameter and 50 cm in height). The objects used in this task were different in shapes, colors, and textures according to Antunes and Biala^60^. The open field and the objects were cleaned between each trial using 70% ethanol to avoid odor trails. Before the experiment day, the animals were allowed to acclimatize to the experimental environment. During habituation, the animals were allowed to freely explore the apparatus without objects for 5 min, once a day for three consecutive days before testing. On the experimental day, animals were submitted to two trials spaced. During the first trial (T1), animals were placed in the area containing the same two identical objects for an amount of time necessary to spend 15 s exploring these two objects in a limit of 4 min. Any mice which did not explore the objects for 15 s within the 4 min period were excluded from experiments. 1 h after exposing to the first trial, the animals were exposed to the second trial (T2). According to this trial, one of the objects presented in the first trial was replaced by an unknown object (novel object). Animals were placed back in the arena for 3 min, the total times which the animals spent to explore or directed the nose within 2 cm of the object while looking at, sniffing, or touching the novel object were recorded and recognized as total exploration time upon novel object.

### Statistical analysis

All data are presented as mean ± SEM values. All data were analyzed by One-way analysis of variance (ANOVA), followed by Tukey’s post hoc test. Probability (*p*) value of less than 0.05 was considered significant. Statistical analysis was performed using SPSS 17.0 software package for Windows.

## Electronic supplementary material

Supplementary file
