# Supplementary material for: Chronic treatment with a smart antioxidative nanoparticle for inhibition of amyloid plaque propagation in Tg2576 mouse model of Alzheimer’s disease
Source: Sci Rep. 2017 Jun 19;7:3785. doi: 10.1038/s41598-017-03411-7 (PMC5476667; doi:10.1038/s41598-017-03411-7)
Supplement: Supplementary file 1 — Supplementary file [file 41598_2017_3411_MOESM1_ESM.pdf]

Supporting Information

Chronic treatment with a smart antioxidative nanoparticle for inhibition of amyloid plaque propagation in Tg2576 mouse model of Alzheimer's disease

*Phetcharat Boonruamkaew, Pennapa Chonpathompikunlert, Long Binh Vong, Sho Sakaue, Yasushi Tomidokoro, Kazuhiro Ishii, Akira Tamaoka, Yukio Nagasaki**

Detailed experimental procedures:

Preparation of the RNP^N:

1. **Synthesis of poly(ethylene glycol) possessing methoxy group at α-end and bromobenzyl group at ω-end [CH_3O-$(CH_2CH_2O)_n$-CH_2PhCH_2Br, *1*] (5 kDa)**

To a 100 g of CH_3O-$(CH_2CH_2O)_n$-H (Wako, Tokyo) in a 500 mL round bottomed flask, 200 mL of dry-THF (Kanto Chemical, Tokyo) was added to completely dissolve the poymer, followed by the addition of 18 mL of butyllithium (1.6 M hexane, Tokyo Kasei) dropwise to convert the end hydroxyl group to the lithium alkoxide. After the addition of p-α,α'-dibromoxylene (25 g), the mixture was stirred at 50 °C for 2 d. The polymer was recovered by repeated precipitations in 2-propanol and dried in vacuo (recovery 90 g, end functionality 94.6%).

2. **Synthesis of PEG possessing methoxy group at α-end and benzyldithiobenzoate group at ω-end [CH_3O-$(CH_2CH_2O)_n$-$CH_2PhCH_2S(C{=}S)Ph$, *2*] (5 kDa)**

To a mixture of dry-THF (40 mL) and carbon disulfide (10 mL, Wako, Tokyo) in a 100 mL round bottomed flask, 10 mL of phenylmagnesium bromide (3 M ether) was added dropwise in ice water bath to form bromomagnesium dithiobenzoate. To a THF (200 mL) of 1 (50 g) in a 500 mL of round bottomed flask, the prepared bromomagnesium dithiobenzoate solution was added and stirred for 2 d at room temperature. The polymer was purified by repeated precipitations in 2-propanol and dried in vacuo (recovery 49 g, end functionality 84.1%).

3. **Synthesis of methoxy-ended-poly(ethylene glycol)-b-poly (chloromethylestyrene) (block copolymer (PEG-b-PCMS, *3*)**

After 20 g of ***2***, 60 mL of chloromethyl styrene and 200 mg of azobisisobutylonitrile (AIBN) were dissolved in toluene (200 mL) in a 500 mL round bottomed flask, the mixture was stirred at 70 °C for 12 h. The obtained polymer was precipitated in 2-propanol and dried in vacuo (34.9 g, the degree of CMS polymerization was 30).

4. **Synthesis of methoxy-ended-poly(ethylene glycol)-b-poly[4-(2,2,6,6-tetramethylpiperidine-N-oxyl)aminomethylstyrene] (PEG-b-PMNT, *4*)**

After 11 g of ***3***, 5 g of 4-amino-2,2,6,6- tetramethylpiperidine-N-oxyl (NH_2-TEMPO), 5 g of ethyldiisopropylamine were dissolved in 50 mL of dimethylformamide (DMF, Wako, Tokyo) in a 100 mL round bottomed flask, the mixture was stirred at 40 °C for 12 h. The obtained polymer was precipitated in 2-propanol and dried in vacuo (9.4 g, TEMPO functionality was 80% determined by ESR).

5. Preparation of RNP^N by *4*

After 5g of ***4*** was dissolved in 200 mL of methanol, the solution was dialyzed against 10 L of water by hollow fiber module (mPES MidiKros® Modules 3 kD ID 0.5 mm D06-

E003-05-N, Spectrum Lab.com, Ritto, Japan). The concentration of the RNP^N solution was adjusted for the purpose.

Supplementary data

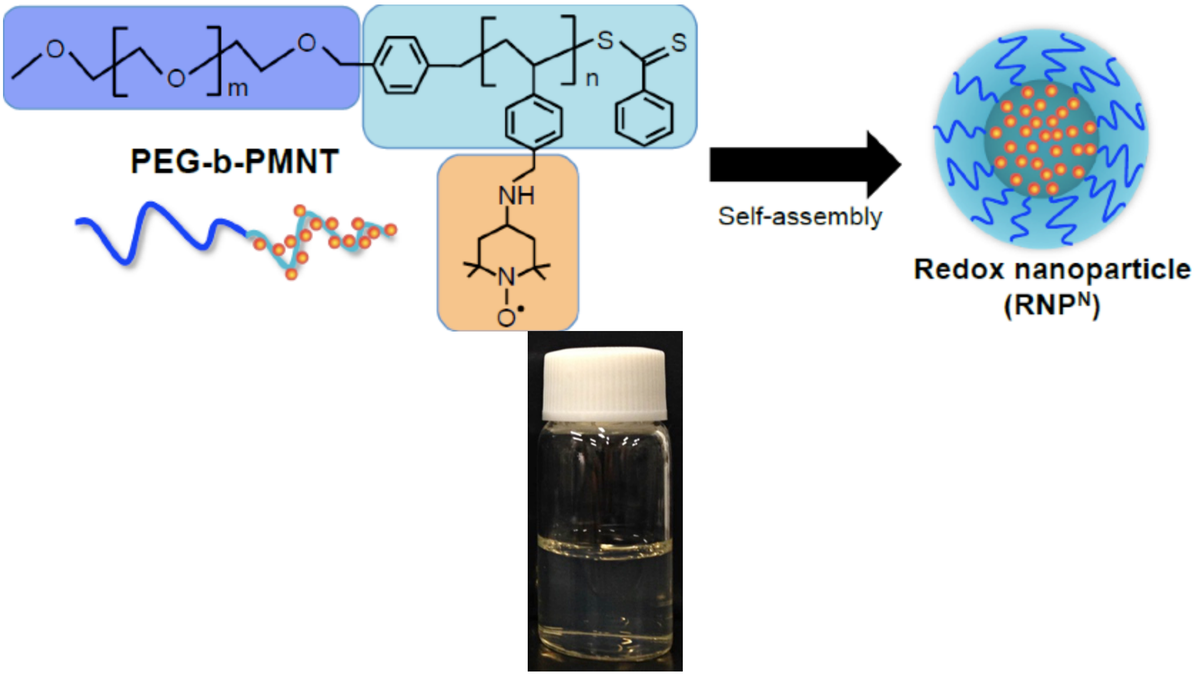

Figure S1. The characteristics of RNP^N solution

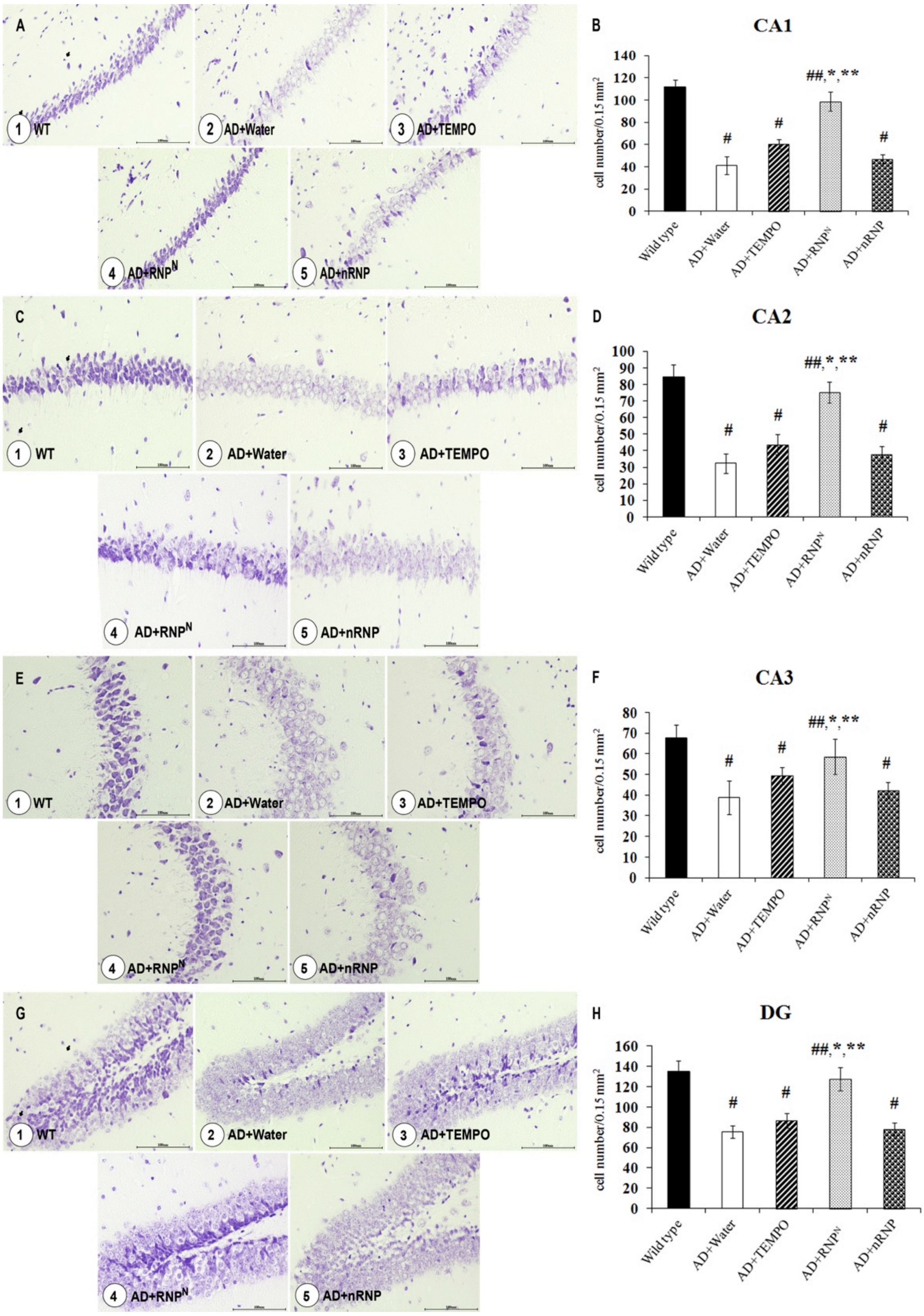

Figure S2. The effect of RNP^N on neuronal densities of hippocampus brain areas; CA1 (A), CA2 (C), CA3 (E) and DG (G). The semi-quantitative analysis of neuronal densities of hippocampus brain areas; CA1 (B), CA2 (D), CA3 (F) and DG (H). Data were expressed as mean ± SEM, # vs wild type group, $p<0.05$; ## vs. AD mice treated with TEMPO, $p<0.05$; * vs. AD mice-treated water group, $p<0.05$; ** vs. AD mice-treated nRNP group, $p<0.05$, n= 5 samples/ group.

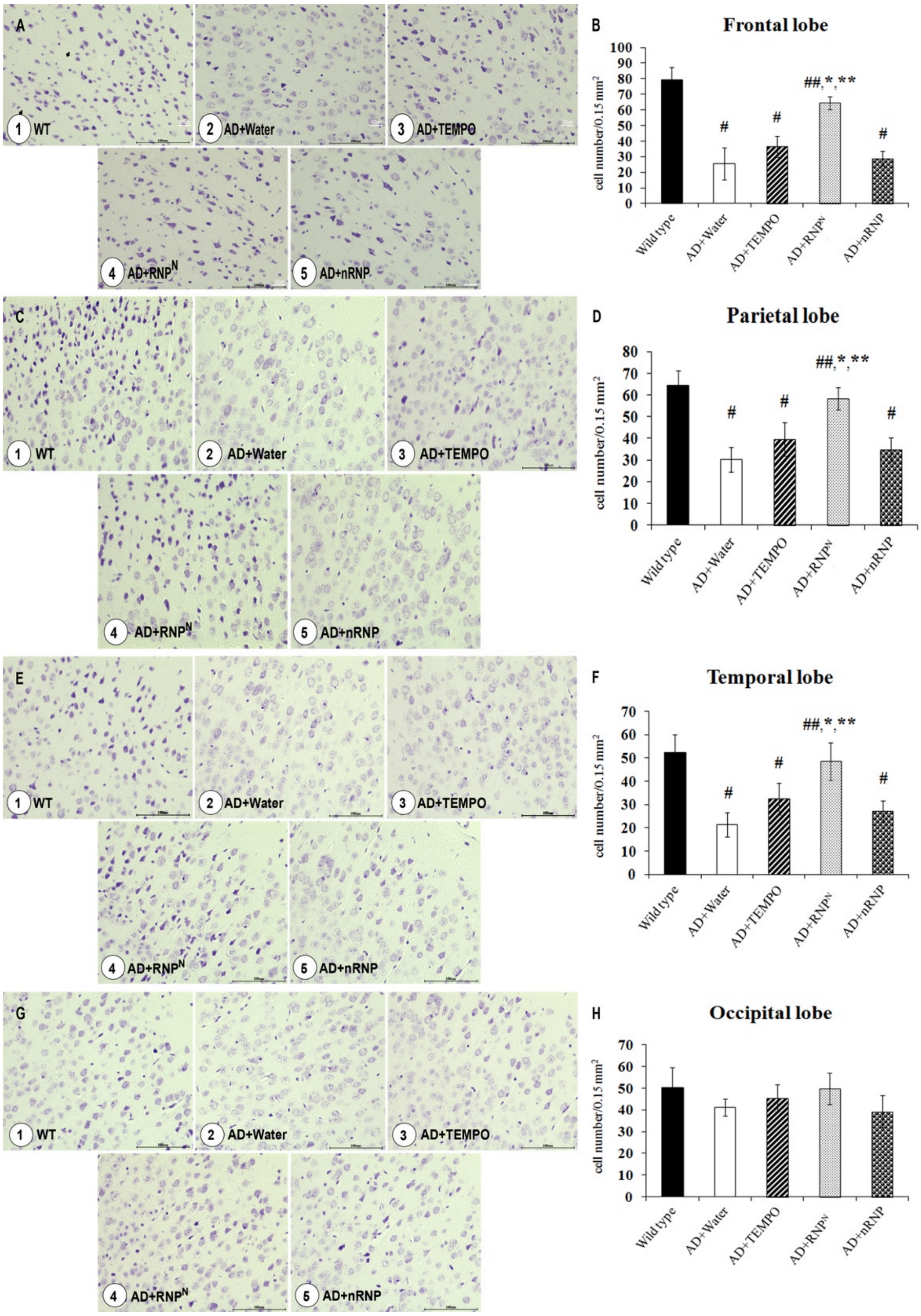

Figure S3. The effect of RNP^N on neuronal densities of cerebral cortex brain areas; frontal (A), parietal (C), temporal (E) and occipital (G). The semi-quantitative analysis of neuronal densities of hippocampus brain areas; frontal (B), parietal (D), temporal (F) and temporal (H). Data were expressed as mean ± SEM, # vs. wild type group, p<0.05; ## vs. AD mice treated with TEMPO, p<0.05; * vs. AD mice-treated water group, p<0.05; ** vs. AD mice-treated nRNP group, p<0.05, n= 5 samples/ group.

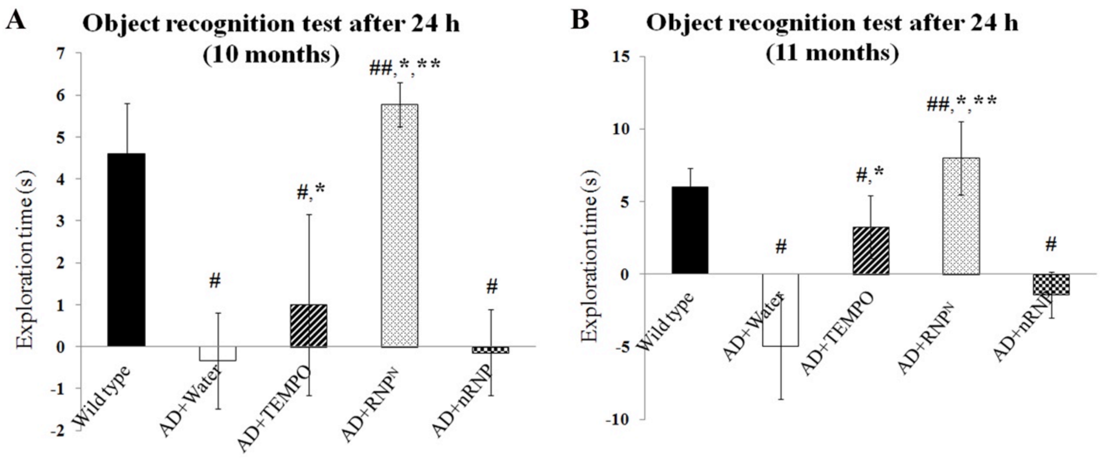

Figure S4. The oral chronic effect of RNP^N –oral free drinking treatment on object recognition test (A-B) after 24 h of administration. Data were represented as mean ± SEM, # vs wild type mice, $p<0.05$; ## vs AD mice treated with TEMPO, $p<0.05$; * vs. AD mice treated with water group, $p<0.05$; ** vs. AD mice treated with nRNP group, $p<0.05$; n = 10 samples/ group.

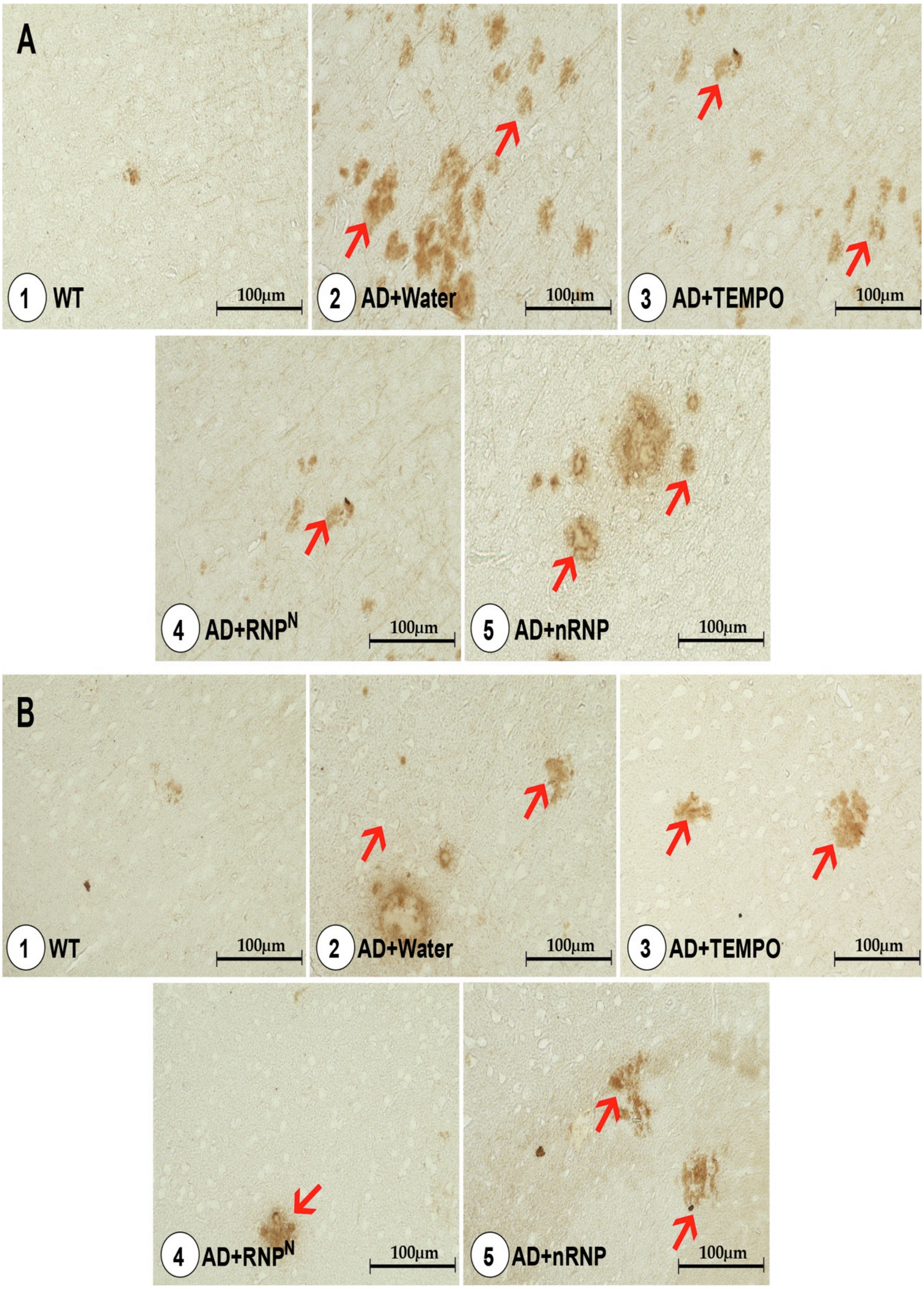

Figure S5. The effect of oral ad libitum drinking of RNP^N on immunohistochemistry staining of Aβ(1-42) (A) and immunohistochemistry staining of Aβ(1-40) (B) of the cerebral cortex of mice brain; 1. wild type group; 2. AD mice group treated with water; 3. AD mice group treated with TEMPO; 4. AD mice group treated with RNP^N; 5. AD mice group treated with nRNP. Scale bar = 100 μm.

Table S1. The quantitative data of ESR spectra (signal intensity) in arbitrary unit (a.u.) from blood and brain of Tg2576 mice after oral free drinking treatment

LMW TEMPO in blood	RNP^{N} in blood	LMW TEMPO in brain	RNP^{N} in brain
4.1819e+003	4.3760e+003	1.9879e+003	2.9187e+003
4.4534e+003	4.5101e+003	1.8891e+003	3.1691e+003
Mean = 4.32e+03	Mean = 4.44e+03	Mean = 1.94e+03	Mean = 3.04e+03
